# Relative quantification of the *recA* gene for antimicrobial susceptibility testing in response to ciprofloxacin for pathogens of concern

**DOI:** 10.1038/s41598-024-52937-0

**Published:** 2024-02-01

**Authors:** Christopher P. Stefan, Candace D. Blancett, Kimberly A. Huynh, Timothy D. Minogue

**Affiliations:** Diagnostic Systems Division, United States Army Medical Research Institute of Infectious Disease, Fort Detrick, MD 21702 USA

**Keywords:** Antimicrobials, Infectious diseases

## Abstract

Antimicrobial resistance (AR) is one of the greatest threats to global health and is associated with higher treatment costs, longer hospital stays, and increased mortality. Current gold standard antimicrobial susceptibility tests (AST) rely on organism growth rates that result in prolonged time-to-answer for slow growing organisms. Changes in the cellular transcriptome can be rapid in the presence of stressors such as antibiotic pressure, providing the opportunity to develop AST towards transcriptomic signatures. Here, we show that relative quantification of the *recA* gene is an indicator of pathogen susceptibly when select species are challenged with relevant concentrations of ciprofloxacin. We demonstrate that ciprofloxacin susceptible strains of *Y. pestis* and *B. anthracis* have significant increases in relative *recA* gene expression after 15 min of exposure while resistant strains show no significant differences. Building upon this data, we designed and optimized seven duplex RT-qPCR assays targeting the *recA* and 16S rRNA gene, response and housekeeping genes, respectively, for multiple biothreat and ESKAPE pathogens. Final evaluation of all seven duplex assays tested against 124 ciprofloxacin susceptible and resistant strains, including Tier 1 pathogens, demonstrated an overall categorical agreement compared to microbroth dilution of 97% using a defined cutoff. Testing pathogen strains commonly associated with urinary tract infections in contrived mock sample sets demonstrated an overall categorical agreement of 96%. These data indicate relative quantification of a single highly conserved gene accurately determines susceptibility for multiple bacterial species in response to ciprofloxacin.

## Introduction

Antimicrobial resistance (AR) is a growing global health crisis. In the United States alone, the economic burden associated with AR infections is estimated around $20 billion annually^1^. However, discovery and approval of clinically relevant novel antibiotics remains perennially under resourced. AR continues to evolve due to drivers such as overuse of prescription antibiotics, with antibiotic therapy deemed inaccurate in almost 50% of cases^2^. This is partly due to the lack of rapid diagnostic tests capable of detecting AR prior to several doses of empiric treatment^3^. Novel diagnostic methods that rapidly guide treatment would significantly improve proper antibiotic stewardship, reducing this global crisis. These diagnostic deficiencies become even more concerning in the context of the purposeful deployment of pathogens or biothreat agents of mass destruction. It is imperative that new tools are developed to identify the susceptibility profile of an organism as rapidly and as close to the site of dissemination as possible.

Current antimicrobial susceptibility tests (AST) mainly target detectable changes in the physical, morphological, or biochemical landscape of bacterial cells in response to antibiotics. Currently, automated broth microdilution instruments approved by the FDA, such as the MicroScan WalkAway, Vitek-2, BD Phoenix, and Senititre, measure growth through changes in turbidity or fluorescence to provide reliable and quantitative results in 4–24 h, depending on growth and antibiotic response rate^4^. Other novel approaches are under development and use optical imaging and light scattering to determine deviations in cell size, shape and number ^5–7^, microcantilevers to measure changes in bacterial mass and micromotions^8,9^, flow-cytometry to determine variations in morphology, viability changes using dyes^10^, and fluctuations in biochemical signatures such as ATP to measure metabolic activity^11,12^. All of these techniques are promising in detecting AR with various pros and cons requiring thorough evaluation prior to clinical use.

Beyond phenotypic changes, clinical labs are rapidly adopting FDA approved genotypic based diagnostics for AR, such as the FilmArray blood culture identification panel^13^. These genetic tests use real-time PCR identification of known AR genes, such as the blaKPC gene found in carbapenem resistant *Enterobacteriaceae*, to determine resistance profiles. While these assays can guide therapeutic decisions, antibiotic susceptibility is much more complex than the presence or absence of the genetic markers^14^. Genotypic AST has also been developed to measure changes in rRNA and DNA signatures to quantify bacterial growth in response to anitbiotics^4,15^. These genotypic assays rely on detecting bacterial growth, limiting their effectiveness for slow growing organisms. A potential mitigation for these issues is the use of RNA transcriptomic signatures to determine susceptibility^16,17^. RNA expression changes have been shown to vary significantly between wild-type and resistant strains leading to the development of several genotypic ASTs^18–20^. These experiments lay the foundation for rapid molecular ASTs designed to detect expression changes in genes conserved across bacteria.

Conserved bacterial pathways are required for survival of antibiotic exposure, including generation of resistance conferring SNPs or induction of a metabolically inactive persistence state^21,22^. The bacterial SOS response is a gene expression pathway activated in response to DNA damage. The fluoroquinolone family of antibiotics inhibit bacterial type II topoisomerases, and thus the bacteria’s ability to control supercoiling, resulting in double-stranded breaks across the genome^23^. These breaks lead to transcriptional regulation triggered by RecA degrading the SOS master repressor, LexA, resulting in the expression of DNA repair and recombination enzymes (RecA and LexA) and DNA polymerases with high error rates^21^. Previously identified SOS signatures have been shown as potential biomarkers of AR in response to ciprofloxacin;^18–20^ however, rapid molecular assays lacked full development and testing across a wide range of organisms.

Fluoroquinolones are considered first-line countermeasures for biothreat agents *B. anthracis* and *Y. pestis*, thus rapid AST is essential to ensure treatments are effective. Similarly, the ESKAPE pathogens made up of *Enterococcus faecium*, *Staphylococcus aureus*, *Klebsiella pneumoniae*, *Acinetobacter baumannii*, *Pseudomonas aeruginosa* and *Enterobacter spp*. are the leading cause of nosocomial infections throughout the world^24^, and are prone to multidrug resistance including fluoroquinolones. In this manuscript, we confirmed significant expression changes in SOS response genes between resistant and susceptible *Y. pestis* and *B. anthracis* strains*.* Consequently, we designed and fully characterized duplex RT-qPCR assays to quantify *recA* expression relative to the 16s rRNA gene for these biothreats as well as several ESKAPE pathogens. Data produced demonstrates RT-qPCR of a single gene in response to the antibiotic ciprofloxacin indicates strain susceptibility across multiple bacterial species.

## Results

### Evaluation of *recA and* SOS gene expression of *B. anthracis* and *Y. pestis* in response to ciprofloxacin

**Figure 1:**
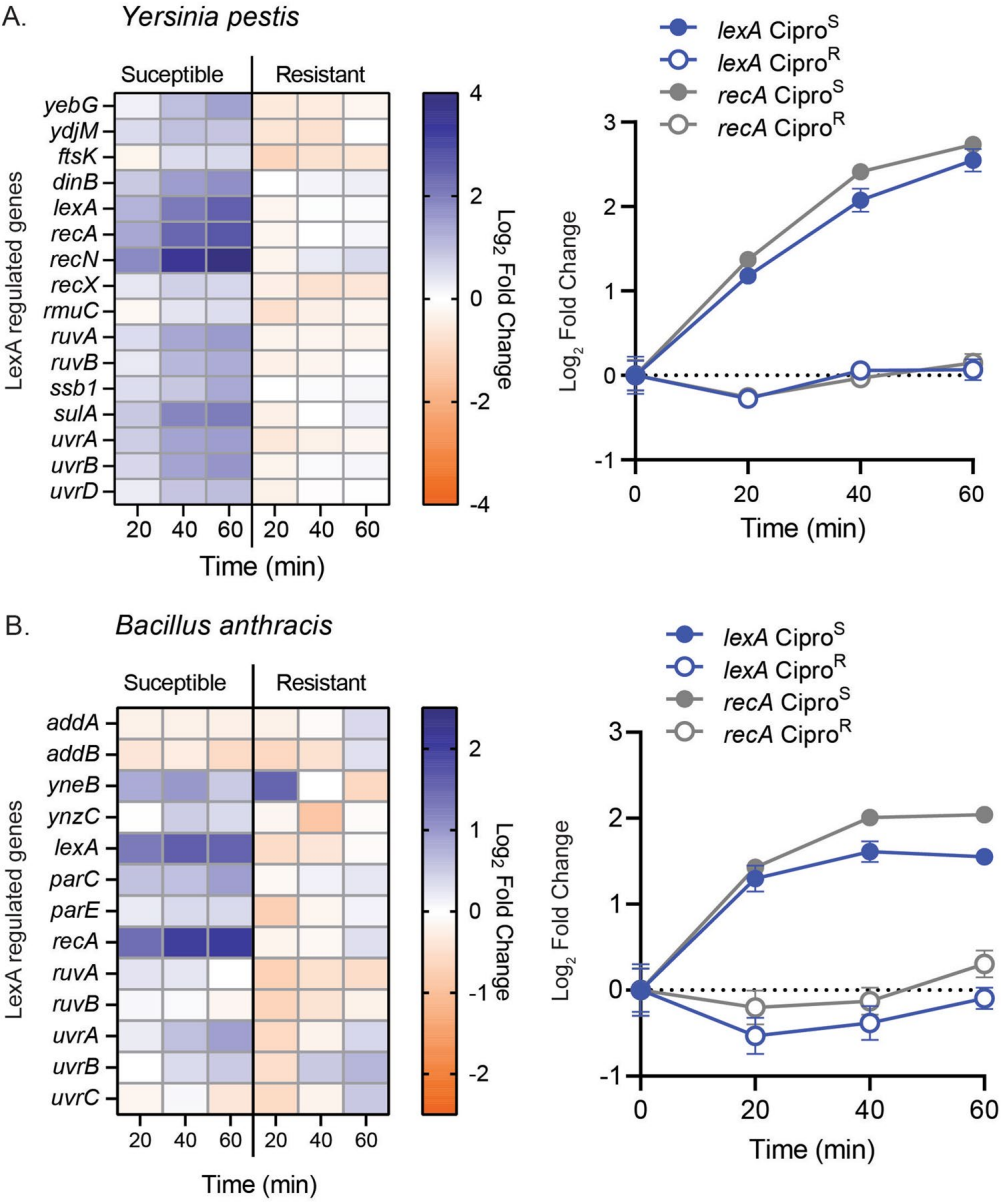
Transcriptomic changes of LexA regulated genes in *Y. pestis* and *B. anthracis* in response to ciprofloxacin. RNA sequencing data shown as a heat map of Log_2_ fold changes in predicted LexA regulated genes for Cipro^R^ and Cipro^S^ strains of (**A**) *Y. pestis* and (**B**) *B. anthracis*, as well as XY plots of Log_2_ fold changes of *lexA* and *recA* against time after 0.25 µg/mL ciprofloxacin exposure. Data points and error bars represent the average and standard deviation respectively of at least three biological replicates.

We evaluated the timing and expression changes of SOS genes in response to ciprofloxacin for select surrogate biothreat pathogens, including *Yersinia pestis* and *Bacillus anthracis* ciprofloxacin resistant (Cipro^R^) and susceptible (Cipro^S^) strains*,* to determine AR gene targets. Differential gene expression was determined using RNA sequencing of strains after the addition of corresponding susceptible breakpoint concentrations of ciprofloxacin, as determined by Clinical Laboratory Standards Institute (CLSI) at various timepoints (Supplemental Figure S1 and S2). Previous literature identified SOS genes as potential markers of AR in *Y. pestis* strains^18–20^; therefore, we focused on genes regulated by the LexA transcription factor as this pathway is evolutionarily conserved and known to turn on rapidly in response to DNA damage. Using RegPrecise, we identified 16 *Y. pestis* and 13 *B. cereus,* a close neighbor to *B. anthracis,* genes regulated by a LexA SOS “box^25^.” For *Y. pestis,* all 16 predicted genes demonstrated an increase in gene expression 60 min postexposure to ciprofloxacin (Fig. 1A, B). Several genes, including *lexA*, *recA*, and *recN,* showed a greater than twofold change in gene expression by 20 min. Similarly, for *B. anthracis*, genes *lexA* and *recA* showed greater than twofold expression changes 20 min after ciprofloxacin addition (Fig. 1C, D). Unlike *Y. pestis*, 50% of the predicted *B. anthracis* SOS genes had no expression change. Cipro^R^ strains demonstrated little to no expression changes in LexA regulated genes after 60 min for either organism (Fig. 1).

**Figure 2:**
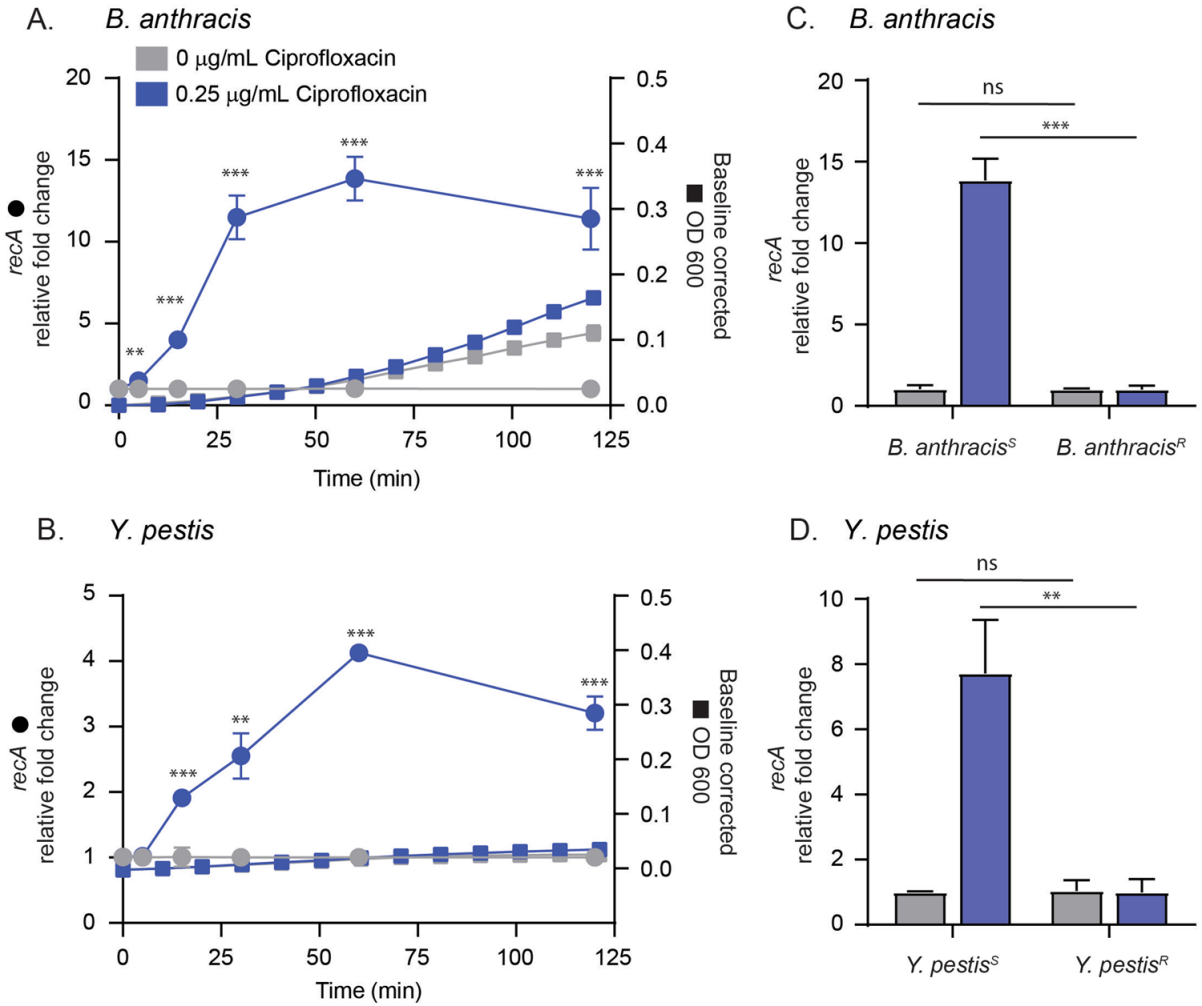
Relative quantification of *recA* in *B. anthracis* and *Y. pestis* in response to ciprofloxacin. Cipro^S^ strains of (**A**) *B. anthracis* and (**B**) *Y. pestis* were treated with and without 0.25 µg/mL ciprofloxacin. Fold change of *recA* (left y-axis, circles) was measured at each timepoint relative to the corresponding no antibiotic control timepoint and the 16S rRNA gene. Optical density (right y-axis, squares) was measured in 10-min intervals across the 2-h time course and data was baseline adjusted to the time 0 timepoint. Data points and error bars represent the average and standard deviation of at least three biological replicates. Asterisks represent statistical significance for *recA* experiments only at indicated timepoints between antibiotic and no antibiotic controls using unpaired t tests. Figure legend in (**A**) is representative of all figures.

We validated relative *recA* expression changes in strains of *B. anthracis* and *Y. pestis* utilizing a duplex RT-qPCR assay targeting *recA* as well as the 16s rRNA gene to serve as an internal, universal housekeeping control. We challenged Cipro^S^ strains with corresponding susceptible breakpoint concentrations of ciprofloxacin and measured relative fold changes over a two-hour time course (Fig. 2). Amplification efficiencies for each duplex RT-qPCR assay were used to determine relative fold changes using a modified ∆∆Cq^26^. The ∆∆Cq method calculates relative gene expression from Cq values obtained from real-time PCR experiments. In this case, relative *recA* fold changes were determined using Cq values obtained from samples treated with and without antibiotics. *B. anthracis* and *Y. pestis* showed statistically significant relative fold changes of *recA*, 4.0 and 1.91 respectively, within 15 min of ciprofloxacin addition, which peaked approximately 60 min after exposure (Fig. 2A, B). Traditional ASTs measure growth rate in the presence and absence of antibiotics and can take several days to produce results. We therefore compared relative *recA* fold expression to optical density changes over time in *B. anthracis* and *Y. pestis* representing a fast and slower growing organism, respectively (Fig. 2A, B). Minimal OD600 changes of 0.2 and 0.0 were seen for *B. anthracis* and *Y. pestis* after 2 h, compared to rapid increases in relative *recA* expression (Fig. 2A, B). To determine if these expression changes were specific to susceptible strains, we challenged Cipro^S^ and Cipro^R^ strains with breakpoint concentrations of ciprofloxacin and measured relative expression after 60 min. Indeed, Cipro^R^ *B. anthracis* and *Y. pestis* strains demonstrated statistically different average *recA* fold changes of 1.0 and 0.99, compared to Cipro^S^ averages of 13.86 and 7.72 (Fig. 2C, D).

**Figure 3:**
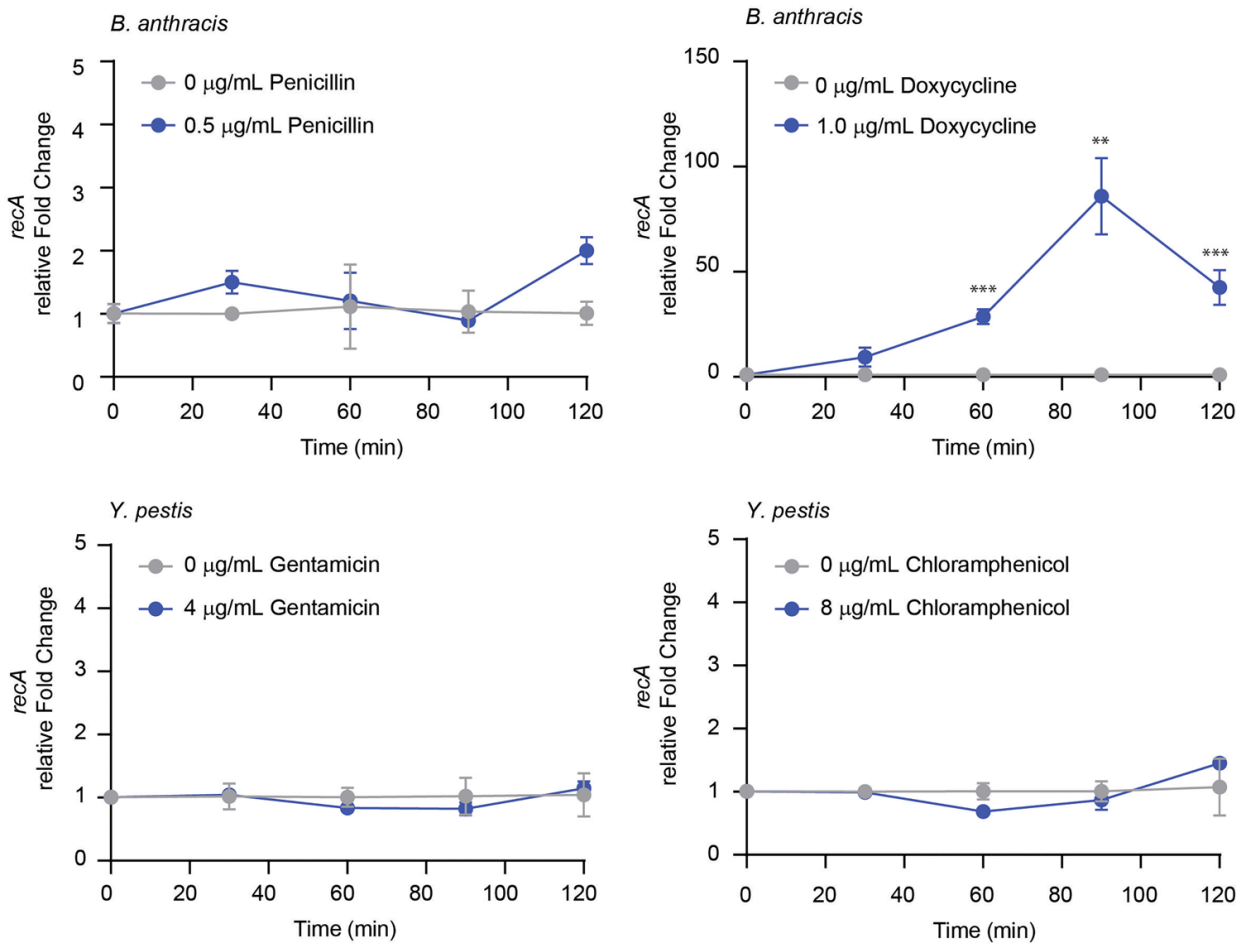
Relative fold change of *recA* in response to clinically relevant antibiotics. *B. anthracis* and *Y. pestis* were treated with and without 0.5 µg/mL penicillin, 1 µg/mL doxycycline, 4 µg/mL gentamicin, and 8 µg/mL chloramphenicol. Fold change of *recA* (left y-axis, circles) was measured at each timepoint relative to the corresponding no antibiotic control timepoint and the 16S rRNA gene. Data points and error bars represent the average and standard deviation of at least three biological replicates. Asterisks represent statistical significance at indicated timepoints between antibiotic and no antibiotic controls using unpaired t tests.

While insult with ciprofloxacin directly results in the creation of double-stranded breaks within DNA, other clinically relevant antibiotics result in the activation of other stress pathways^27,28^. We tested clinically relevant antibiotics penicillin, doxycycline, gentamicin, and chloramphenicol on susceptible strains of *B. anthracis* and *Y. pestis* (Fig. 3) to determine if *recA* is upregulated in response to these various antibiotic families. We showed no significant relative *recA* fold changes in response to penicillin, gentamicin, and chloramphenicol over a two-hour time course; however, doxycycline induced significant changes to *recA* expression. We observed a sevenfold relative change after 30 min with a peak relative fold change after 90 min. These results suggest doxycycline as another antibiotic to evaluate for future testing.

### Design of real-time RT-qPCR assays to measure relative fold changes of *recA* gene in multiple bacterial species

The SOS pathway is not 100% conserved throughout bacteria, suggesting regulatory and response genes vary from species to species^29^. Along with the *B. anthracis* and *Y. pestis* assays described above, we designed duplex RT-qPCR assays targeting the *recA* gene for several ESKAPE pathogens to determine conservation of relative *recA* expression across multiple species (Table 1). Optimization of these assays, as well as prior *B. anthracis* and *Y. pestis* assays, included linearity testing, efficiency calculations, and reproducibility at defined assay concentrations (Table 2). Assay efficiencies represent the ability of an assay to double the number of targets during each cycle of replication, therefore a 100% efficient assay has an amplification factor of 2. The average amplification factor across all organisms, calculated from serially diluted extracted nucleic acid, were 2.03 and 1.92 for the *recA* and 16S rRNA gene respectively. Furthermore, nucleic acid extracted from a defined assay concentration of 0.5 McFarland showed comparable means, standard deviations, and coefficients of variance across 60 replicates for each organism (Table 2).

**Table 1:** Primers and Probes used throughout manuscript.

Organism	Gene	Primer F	Primer R	Probe	Quencher
*Bacillus anthracis*	*recA*	CCGTGTTGCGGAAGTTGAT	CACCGCTCTTTTGAACGATATC	AACGTCTACAGCTTCGTAAA	MGB
*Yersinia pestis*	*recA*	CGCTTCTGTACGTTTGGATATCC	CTCCCCACGACCACATCAC	CGTATTGGTGCAGTAAA	MGB
*Acinetobacter baumannii*	*recA*	AAATACGGTTATGCGTCTTGG	GTCTAAAGTTAAAGAACCTGTAGATACG	ACTGTTCAAGCAGTTGAA	MGB
*Staphylococcus aureus*	*recA*	AAAGTTGGTGTTATGTTCGGTAATC	CTTGTCCTTGTTTAAGCTGTTCTG	ACTACACCAGGTGGACGTG	MGB
*Klebsiella pneumoniae*	*recA*	GCGGCGCGTATGATGAG	AAGATCAGCAGCGTGTTGGA	TGGCGGGTAACCTG	MGB
*Escherichia coli*	*recA*	GCTATCGACGAAAACAAACAGAAA	CATCCATGGAACGGTCTTCAC	TTGGCGGCAGCACT	MGB
*Enterobacter cloacae*	*recA*	TCGACGAAAACAAACAGAAAGC	TGATGGAGCCTTTACCGAATTG	TTGGCGGCAGCACT	MGB
*Universal*^42^	16S	CCTACGGGDGGCWGCA	GGACTACHVGGGTMTCTAATC	CAGCAGCCGCGGTA	MGB

**Table 2:** Results from verification of duplex RT-qPCR assays.

Organism	Amplification factor		Assay efficiency		*recA *confirmation				*16S *confirmation			
	*recA*	16S	*recA* (%)	16S (%)	#	Cq mean	Cq std	Cq CV (%)	#	Cq mean	Cq std	Cq CV (%)
*Bacillus anthracis*	2.10	1.92	110	92	60/60	30.82	0.27	0.87	60/60	20.39	0.21	1.0
*Yersinia pestis*	2.03	1.93	103	93	60/60	22.99	0.09	0.39	60/60	12.33	0.49	4.0
*Acinetobacter baumannii*	2.01	1.93	101	93	60/60	25.14	0.05	0.22	60/60	13.21	0.12	0.88
*Staphylococcus aureus*	2.01	1.96	101	96	60/60	25.11	0.44	1.8	60/60	15.71	0.45	2.8
*Klebsiella pneumoniae*	2.02	1.85	102	85	60/60	26.47	0.35	1.3	60/60	13.45	0.38	2.8
*Escherichia coli*	2.03	1.94	103	94	60/60	25.23	0.52	2.1	60/60	13.34	0.71	5.4
*Enterobacter cloacae*	2.03	1.96	103	96	60/60	23.62	0.08	0.32	60/60	11.86	0.32	2.7

### RT-qPCR *recA* duplex assay performance in culture and contrived complex samples

We used these optimized and characterized assays to determine initial performance metrics in broth culture medium as well as contrived complex samples. We challenged 124 Cipro^S^ and Cipro^R^ organisms, including Tier 1 agents (Supplementary Table S1), with corresponding breakpoint concentrations of ciprofloxacin: 12 *Acinetobacter baumannii,* 12 *Enterobacter cloacae,* 11 *Escherichia coli,* 12 *Staphylococcus aureus,* 12 *Klebsiella pneumoniae*, 46 *Bacillus anthracis* and *19 Yersinia pestis* strains (Table 3, Supplemental Figure S3). For Cipro^S^ strains, relative *recA* fold changes were highly variable between species. For instance, *Y. pestis* Cipro^S^ strains had an average relative expression of 3.29 while *E. coli* Cipro^S^ strains showed an average of 25.56 (Table 3). In contrast, the relative fold change for Cipro^R^ strains was consistently near 1. When combined, the average fold change for Cipro^S^ and Cipro^R^ strains was 7.83 and 1.12 respectively (Table 3). To define initial performance metrics, major errors (ME) were defined as false-resistant calls while very major errors (VME) were defined as false-susceptible calls. The total number of susceptible and resistant strains were used as the denominator for ME and VME rates. Error rates were determined based on a universal relative *recA* fold change threshold of 1.54. This threshold was optimized to obtain the greatest balance between sensitivity and specificity based on the current dataset. Because no training set was used to calculate a threshold, a larger independent sample set would be required for final determination of diagnostic metrics. Using a cutoff of 1.54 across all species resulted in overall categorical agreement of 97% when compared with micro-broth culture and ME rates of 1.6% and VME rates of 4.9%.

**Table 3:** Assay performance in culture and contrived complex samples.

Organism	Antibiotic, µg/mL	AMR	# strains	Mean	Std. dev	Min	Max	Lower 95% CI	Upper 95% CI	VME^a^	ME^a^
Cell culture											
* Klebsiella pneumoniae*	1	Susceptible	6	11.2	4.01	6.03	16.71	6.95	15.4	0	0
		Resistant	6	1.10	0.17	0.94	1.39	0.93	1.28	0	0
* Staphylococcus aureus*	1	Susceptible	6	5.90	1.73	3.53	7.86	4.09	7.72	0	0
		Resistant	6	1.11	0.30	0.68	1.51	0.79	1.42	0	0
* Escherichia coli*	1	Susceptible	6	25.6	20.4	9.94	55.5	9.94	55.5	0	0
		Resistant	6	1.11	0.14	0.91	1.31	0.91	1.31	0	0
* Acinetobacter baumannii*	1	Susceptible	3	8.58	1.06	7.56	9.68	5.94	11.2	0	0
		Resistant	9	1.06	0.29	0.55	1.25	0.83	1.28	0	0
* Enterobacter cloacae*	1	Susceptible	6	14.6	10.4	5.52	32.8	3.66	25.4	0	0
		Resistant	6	1.15	0.28	0.74	1.47	0.86	1.44	0	0
* Bacillus anthracis*	0.25	Susceptible	27	5.16	2.67	1.56	0.67	4.10	6.21	0	0
		Resistant	19	1.18	0.42	12.1	2.42	0.98	1.38	2	0
* Yersinia pestis*	0.25	Susceptible	11	3.29	1.67	0.69	5.87	2.16	4.41	0	1
		Resistant	8	1.08	0.34	0.60	1.57	0.79	1.36	1	0
Total	Variable	Susceptible	63	7.83	8.17	0.69	55.5	4.73	7.28	3	0
		Resistant	61	1.12	0.31	0.55	2.43	1.01	1.21	0	1
Contrived mock urine culture											
* Escherichia coli*	1	Susceptible	5	14.8	8.73	3.35	26.4	3.96	25.7	0	0
		Resistant	7	0.95	0.17	0.61	1.15	0.79	1.11	0	0
* Klebsiella pneumoniae*	1	Susceptible	6	6.62	1.7	4.32	9.43	4.75	8.48	0	0
		Resistant	6	1.37	0.68	1.03	2.76	0.66	2.09	1	0

^a^VME and ME based on cutoff threshold of relative *recA* fold-change of 1.54.

To test the use of these assays beyond broth culture, we explored testing directly from a complex matrix with high bacterial burdens. Until recently, fluoroquinolones were considered a first-line treatment option for simple cystitis urinary tract infections (UTI), and are still used in complicated UTIs and as second line therapy in cases of first-line resistance^30^. We tested isolates of common UTI organisms directly from overnight growth in urine without secondary culture to test if the duplex RT-qPCR *recA* assays could determine susceptibility directly from complex matrix. In total, testing 24 strains including 12 *E. coli* and 12 *K. pneumoniae* resulted in only one false susceptibility call (Table 3). Of these 24 strains, 12 were replicated from broth culture testing above. Comparison of these strains between the two experiments demonstrated a Pearson’s value of 0.91, indicating high correlation between experiments and strains (Supplemental Fig. S4). These results suggest susceptibility calls can be performed from primary samples after dilution in broth culture, eliminating the need for secondary culture, significantly reducing time-to-answer compared to gold-standard growth assays.

## Discussion

Molecular methods targeting genetic elements, such as resistance genes, have gained popularity; however, the presence or absence of genes does not always correlate with resistance^14^. Culture methods are labor intensive with significant variations in time-to-answer due to variable organism growth rates. Automated broth microdilution instruments significantly reduce hands-on time; however, these instruments are large and not conducive for point-of-need settings. It is imperative to develop rapid ASTs that are functional as close to point-of-need as possible, especially in the context of AR biothreat pathogens. In this manuscript we focus on the development of molecular ASTs to determine fluoroquinolone susceptibility given the recommended guidance for clinical management of biothreat agents in the event of an intentional release. Specifically, a 60-day oral treatment regimen of ciprofloxacin for cutaneous anthrax, a multi-drug combination for systemic anthrax syndromes^31^, and a 7 day course for postexposure prophylaxis for *Y. pestis*^31^. In the biothreat space, the continued development of novel AST methods ensures we have effective countermeasures towards priority pathogens; however, in a broader context, AST methods that expand beyond these pathogens are needed to help combat the rise in AR.

Bacteria have evolved several signaling pathways that respond rapidly to cellular stressors such as antibiotics^27^. For instance, the envelope stress response is activated in the presence of cell wall and protein synthesis inhibiting antibiotics, such as glycopeptides and aminoglycosides^27^, while the oxidative stress pathway responds to the production of reactive oxygen species created in the presence of bactericidal antibiotics such as β-lactams, aminoglycosides, and quinolones^28^. Another conserved^29^ stress pathway, the SOS response and its chief regulator RecA, is activated in the presence of excessive single-stranded DNA originating from endogenous or exogenous factors, such as stalled replication forks or UV light and antibiotics, respectively^23^. Fluoroquinolones activate the SOS response by directly inhibiting DNA gyrase and topoisomerase IV, resulting in double-stranded DNA breaks^17,20^. To highlight the conservation of the SOS pathway, when challenged with ciprofloxacin we saw increases in relative *recA* fold changes across seven different bacterial species, and 62 of the 63 total susceptible strains tested within this manuscript. We also tested other relevant antibiotics as the creation of reactive oxygen species via sublethal concentrations of bactericidal antibiotics has been shown to activate the SOS pathway^21,32,33^. We saw no significant change in *recA* induction for *B. anthracis* and *Y. pestis* strains when challenged with bactericidal antibiotics penicillin or gentamicin; however, doxycycline, a bacteriostatic protein synthesis inhibitor, resulted in a drastic relative fold change in *B. anthracis.* While not exhaustive, these results indicate differential regulation of the SOS response across multiple antibiotic classes and should be further explored as potential targets for AST development.

While highly conserved, functionality of the RecA protein and SOS system in response to stimuli is not preserved throughout bacterial species. For instance, organisms like *Neisseria gonorrhoeae* have functional RecA proteins but lack an SOS system^34^. Even if activated, our data show highly variable relative expression across bacterial species (Table 3) complicating the use of transcriptomic signatures for AST development. These variations likely result from species-specific factors including differential expression of influx/efflux pumps, presence or absence of various stress pathways, or even differences in primary target^23^. For example, it has been shown that fluoroquinolones mostly target DNA gyrase in gram-negative organisms, while topoisomerase IV is the preferential target in gram-positive organisms^23^. Beyond inter-species variation, our data also demonstrate reproducible strain-to-strain variations in relative *recA* expression levels. For instance, *E. coli* strain AR0069 demonstrated a reproducible 2× increase in expression compared to strain AR0089 (Supplemental Figure S4). While both are sensitive to ciprofloxacin, these strains have differential susceptibility profiles to other antibiotics that could affect the global stress response and thus *recA* expression.

Differences in MICs may also play a significant role in intra-species variations. We evaluated susceptible breakpoint concentrations within this manuscript; however, three breakpoint concentrations including susceptible, resistant, and intermediate^35^, are listed within the CLSI guidelines. While we evaluated a range of MICs, none were within the intermediate range; therefore, assay performance remains unknown. No strong correlation was identified when comparisons between MICs and relative fold changes were evaluated (Supplemental Fig. S4); however, a more in-depth analysis with more strains at or near the various breakpoints is warranted. In totality these data highlight that several factors need to be considered, not just species-to-species, but strain-to-strain, when developing molecular AST targeting transcriptional changes.

One challenge associated with developing molecular ASTs is how to define a definitive threshold to measure key diagnostic parameters. We utilized a threshold of 1.54 relative fold change to define preliminary metrics. This threshold was based on receiver operating curve (ROC) analysis of all datapoints and resulted in optimal sensitivity and specificity metrics. Utilizing all the available datapoints to define a threshold will skew performance metrics; however, the required number of susceptible and resistant strains essential to define a true threshold based on a subset, or training set, were not available for testing. Future work on larger sample-sets could more granularly define a threshold for each species given the clear differential response between resistant and susceptible strains (Table 3). There is also the potential to maintain the use of a universal threshold based on the consistent relative *recA* fold change identified among resistant strains. While susceptible strains demonstrated high variations, as described above, the mean of all 61 combined resistant strains was 1.12 with a 95% confidence interval ranging from 1.04 to 1.20, indicating little change in expression (Table 3). Reproducibility and robustness studies would need to be completed before defining a true threshold for diagnostic use; however, based on the low variability seen in resistant strains, a universal threshold could be applied if adequate strains cannot be tested to define species specific cutoffs.

The speed and sensitivity of molecular testing compared to traditional methods drove development of several PCR based assays for susceptibility testing ^18–20,36–38^. Amplification based approaches allow resolution of minute changes in target copy numbers compared to less sensitive optical density measurements. Novel methods utilizing high-throughput microscopy, cytometry, or mass spectrometry require complex instrumentation; comparatively, PCR technologies can be miniaturized, and several instruments are usable in austere settings^39,40^. We limited the data for this manuscript to reference lab instrumentation; however, bridging experiments would permit utilization on more point-of-need devices, including portable real-time PCR instruments such as the Biomeme platform^39,40^. Similarly, differential extraction methods or “extractionless PCR” could be utilized to rapidly prepare nucleic acids for amplification. Most importantly for impacting patient treatment, using the amplification approaches here would allow the identification of molecular changes occurring well before gross phenotypic variations could be quantified. The continuous advancement in molecular diagnostic technologies combined with the speed and sensitivity of detection afforded by the described *recA* assays warrants further exploration for its use in point-of-need AST development.

Several molecular AST methods quantify changes in gene transcripts as cells grow, mimicking broth microdilution tests^36–38^. Specifically, the MAPt method applies sample directly to micro-agar wells containing serially diluted antibiotic and measures growth using quantitative PCR. As this method is still based on growth, exposure times to determine susceptibility for Tier-1 agents are significantly increased for slow-growing organisms^36^. The MAPt method, however, is independent of starting bacterial concentrations and thus can be applied directly to primary samples such as whole blood or environmental samples, forgoing primary enrichment steps. Currently, the *recA* assay requires primary enrichment and results in qualitative calls of susceptibility due to the use of susceptible breakpoint concentrations of antibiotic defined by strict starting cellular concentrations. Future efforts need to focus on varying concentrations of cell/antibiotic concentrations in complex matrices, especially from whole blood, to define a molecular MIC and determine if primary enrichment is required. This data would contribute to the usefulness of the *recA* assays in more routine point-of-need settings where complex culture systems are not present.

Taken together, the data presented demonstrates the feasibility of rapid and reproducible molecular AST assays utilizing relative quantification of the *recA* gene across multiple genera including major ESKAPE pathogens and biothreat agents. Importantly, we demonstrated categorical agreements to broth microdilution methods utilizing the relative quantification of a single highly conserved gene. This conservation could be utilized to develop *recA* assays toward conserved nucleic acid regions amplifying multiple genera within select families. For instance, during assay development, alignment of several Enterobacteriaceae family members demonstrated conserved nucleic acid regions that could be targeted by a pan-*recA* assay (Supplemental Fig. S5). In a contrasting strategy, highly specific assays could be employed that determine susceptibility and classify the organism in a single easy to use assay. Future efforts should explore expanding assay panels for these applications as well as transitioning assays toward more point-of-need devices focusing on testing directly from complex matrices.

## Material and methods

### Bacterial strains and experimental setup

Bacterial strains used in this study are included in Supplementary Table S1. Several strains were acquired from the CDC and FDA antibiotic resistance isolate bank^41^ Glycerol stocks were plated on sheep’s blood agar plates (Thermo Fisher Scientific, Waltham, MA, Catalog #R01200), grown overnight at 37 °C, or 28 °C for *Y. pestis*, and selected for recovery the following day in tryptic soy broth (TSB) (Thermo Fisher Scientific, Catalog #R112732). Cells were recovered in TSB for approximately 3 h then diluted to a McFarland standard of 0.5 using the DensiChek Plus (Biomerieux, Marcy-l'Étoile, France, Catalog #21255). One milliliter of diluted cells with or without indicated concentrations of ciprofloxacin were incubated in 5 mL Eppendorf tubes (Eppendorf, Hamburg, Germany, Catalog # 0030119401) at 37 °C in a shaking incubator for 60 min (or indicated time). For mock clinical samples, cells were cultured overnight in urine (BioreclamationIVT, Baltimore, MD). Cultures were normalized to 0.5 McFarland and diluted 1:10 in TSB for a final volume of 1 mL prior to antibiotic insult. For sequencing and primary time-course experiments 2 volumes of RNAprotect Bacteria Reagent (Qiagen, Valencia, Ca, Catalog #76104) was added to each tube and incubated at room temperature for 5 min at indicated time points. For all reactions tubes were centrifuged at 5000×*g* for 10 min. The supernatant was decanted, and pellets were extracted or stored at −80 °C until processing.

### Cell lysis and RNA extraction

Cell pellets were extracted by two methods. For sequencing and time course experiments, pellets were resuspended in 100µL 1xTE containing 15 mg/mL lysozyme (Sigma-Aldrich, St. Louis, MO, Catalog # 10837059001) and 10 µL Proteinase K (Qiagen, Catalog #RP107B-1). Suspended pellets were incubated at room temperature for 10 min. Then, 700 µL of buffer RLT containing 7 µL of β-mercaptoethanol (BME)  (Qiagen, Catalog #79216) was added to suspended pellets along with approximately 100 µL of 0.5 mM glass beads and cells were incubated at 95 °C for 5 min, bead beaten for 10 min, then centrifuged ≥ 8000×*g* for 30 s. 760 µL of supernatant was added to new 2 mL Eppendorf tubes containing 590 µL of 80% ethanol. Samples were then extracted utilizing the RNeasy kit protocol and an on-column DNase treatment according to manufacturer’s protocols (Qiagen, Catalog #74004). For assay AST experiments cell pellets were resuspended in 500 µL buffer RLT containing 5 µL BME (Sigma-Aldrich, Catalog #444203), 100 µL of 0.5 mM glass beads, and bead beat for 5 min. A total volume of 400 µL was extracted on the EZ-1 Advanced XL robotic extraction instrument using the Viral RNA mini kit v2.0 (Qiagen, Catalog #955134) according to manufacturer’s instructions. Purified RNA was stored at −80 °C.

### Total RNA sequencing and analysis

Extracted RNA was quantified using the Qubit 2.0 Fluorometer and Qubit RNA HS assay (Thermo Fisher Scientific, Catalog #Q32852). RNA was spiked with the ERCC control (Thermo Fisher Scientific, Catalog # 4456740), an external RNA control, and input into the QIAseq FastSelect—5s/16s/23s kit (Qiagen, catalog #335927) to remove bacterial ribosomal RNA prior to sequencing. Sequencing libraries were prepared using the QIAseq Stranded total RNA library kit according to manufacturer’s instructions (Qiagen, Catalog #180745). Libraries were quantified using the High Sensitivity D1000 kit on the 4200 Tapestation System (Agilent Technologies, Santa Clara, CA, Catalog # 5067–5585). Four barcoded libraries were combined and diluted to a 4 nM library pool, denatured with NaOH, and further diluted to a final library concentration of 12 pM prior to sequencing. Pooled libraries were sequenced using the MiSeq Instrument and MiSeq reagent kit version 3–150 cycles (Illumina, San Diego CA, Catalog #MS-102-3001). FASTQ files were imported into CLC Genomics workbench and analyzed using the RNA-Seq Analysis workflow. Paired sequencing reads were mapped to *B. anthracis* GCF_000008445.1_ASM844v1_genomic and *Y. pestis* GCF_000222975.1_ASM22297v1 reference sequences using the following mapping setting: mismatch cost (2), insertion cost (2), deletion cost (3), length fraction (0.8), and similarity fraction (0.8). Each timepoint was composed of three independent biological replicates. Paired reads were counted as two when calculating transcripts per million (TPM). Treated samples were compared to timepoint 0 untreated controls for differential analysis. Differentially expressed genes and ANOVA analysis were determined using JMP Genomics 8 (JMP, Campus Drive Cary, NC).

### cDNA synthesis and real-time PCR

cDNA was made using the SuperScript IV VILO master mix with ezDNase Enzyme kit according to manufacturer’s instructions (Thermo Fisher Scientific, Catalog #11756050). Briefly, 8 µL of extracted RNA was added to 10× ezDNAse buffer and ezDNase enzyme for a final volume of 10 µL. Reactions were incubated at 37 °C for 5 min at which time 4 µL of SuperScript IV VILO master mix was added along with water to a final volume of 20 µL. Reactions were gently mixed and incubated at 25 °C for 10 min, 50 °C for 10 min, 85 °C for 5 min, and then held at 4 °C until removal. Reactions were diluted 1:10 with water and then stored at − 20 °C until use.

Primers and TaqMan-minor groove binder (MGB) probe pairs, shown in Table 1, were designed using Primer Express version 3.0.1 (Applied Biosystems, Foster City, CA). Primers and probes were ordered from Thermo Fisher Scientific. Assay conditions were determined empirically through optimization of primer/probe and MgCl_2_ concentrations. Final reaction conditions were as follows: 1× buffer with 3 mM final MgCl_2_ concentration (Biofire Diagnostics, Salt Lake City, UT, Catalog # 1770), 200 nM dNTPs (Biofire, Catalog # 1774), 4.0 µM forward and reverse recA primers, 4 µM forward and reverse 16S primers^42^, 0.1 µM recA-FAM probe, 0.1 µM BactQuant-VIC probe, 0.2 µL of Platinum Taq (Thermo Fisher Scientific, Catalog # 15966025), 5 µL cDNA, and water to a final volume of 25 µL. Assays were run on the LightCycler 480 (Roche Applied Science, Indianapolis, IN) using the following cycling conditions: 95 °C for 2 min (1 cycle); 95 °C for 15 s, 60 °C for 30 s, 68 °C for 30 s (45 cycles).

### Supplementary Information

Supplementary Figures.

Supplementary Table S1.

## Data Availability

Sequencing datasets generated and/or analyzed during the current study are available in the Gene Expression Omnibus (GEO) repository, GSE241489.
