# Supplementary material for: Relative quantification of the recA gene for antimicrobial susceptibility testing in response to ciprofloxacin for pathogens of concern
Source: Sci Rep. 2024 Feb 1;14:2716. doi: 10.1038/s41598-024-52937-0 (PMC10834403; doi:10.1038/s41598-024-52937-0)
Supplement: Supplementary file 1 — Supplementary Figures. [file 41598_2024_52937_MOESM1_ESM.docx]

**Relative quantification of the *recA* gene for antimicrobial susceptibility testing in response to ciprofloxacin for pathogens of concern**

Christopher P. Stefan, Candace D. Blancett, Kimberly Huynh, Timothy D. Minogue

Contents:

Supplementary Figure S1: Transcriptomic analysis of Cipro^S^ and Cipro^R^ strains of *B. anthracis* in response to susceptible breakpoint concentrations of ciprofloxacin.

Supplementary Figure S2: Transcriptomic analysis of Cipro^S^ and Cipro^R^ strains of *Y. pestis* in response to susceptible breakpoint concentrations of ciprofloxacin.

Supplementary Figure S3: Assay performance utilizing *recA* fold changes to determine strain susceptibility.

Supplementary Figure S4: Correlation of relative recA fold amongst strains and MICs

Supplementary Figure S5: Alignment of recA gene for several members of the Enterobacteriaceae family.


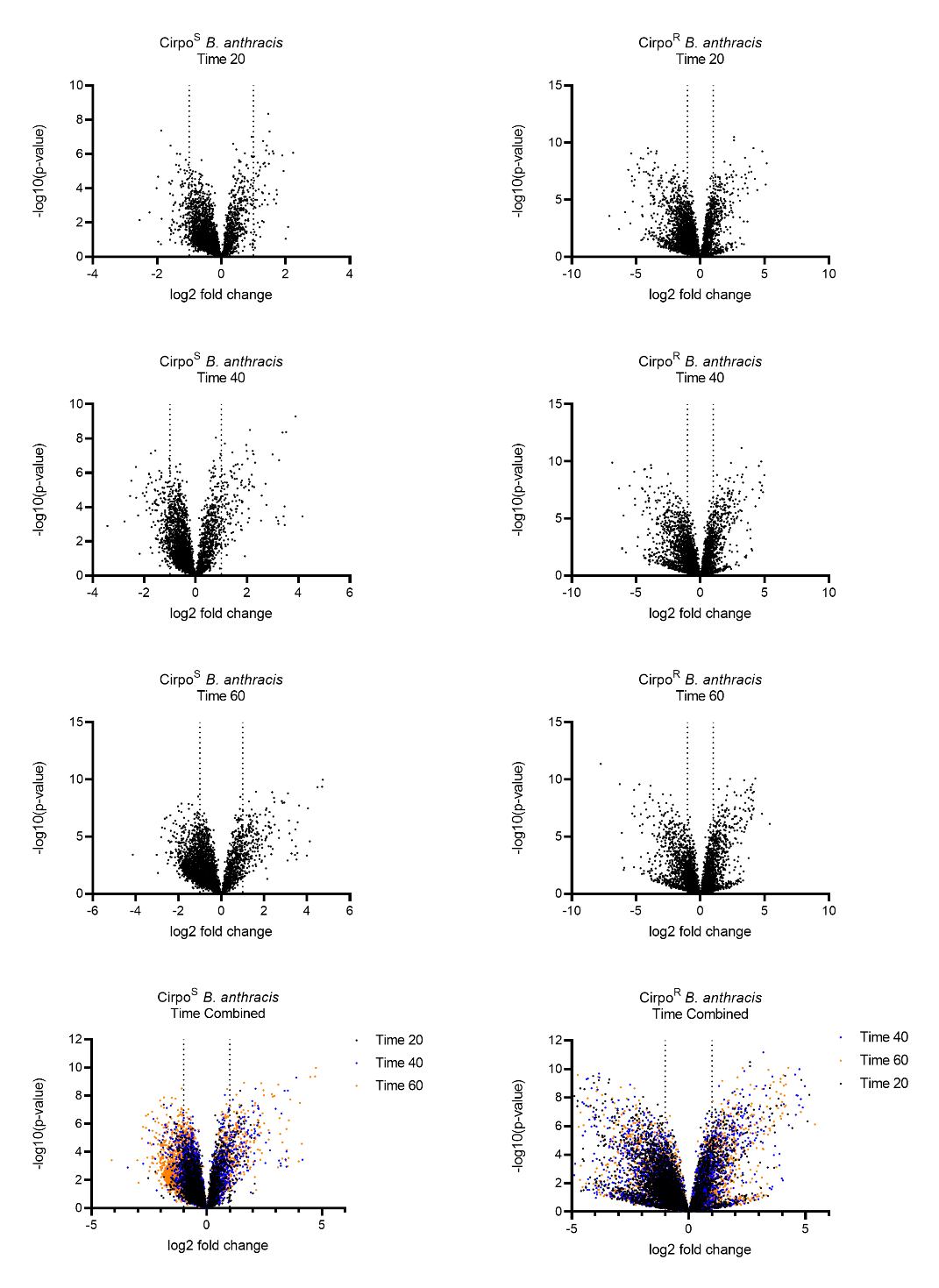


**Supplementary Figure S1: Transcriptomic analysis of Cipro^S^ and Cipro^R^ strains of *B. anthracis* in response to susceptible breakpoint concentrations of ciprofloxacin.** Volcano plots of RNA sequencing data showing log_2_ fold changes vs -log10(p-value) in Cipro^R^ and Cipro^S^ strains of *B. anthracis* across multiple timepoints after exposure to 0.25 µg/mL ciprofloxacin. Data is representative of RNA sequencing data from three biological replicates treated at each time point compared to time 0 untreated controls.


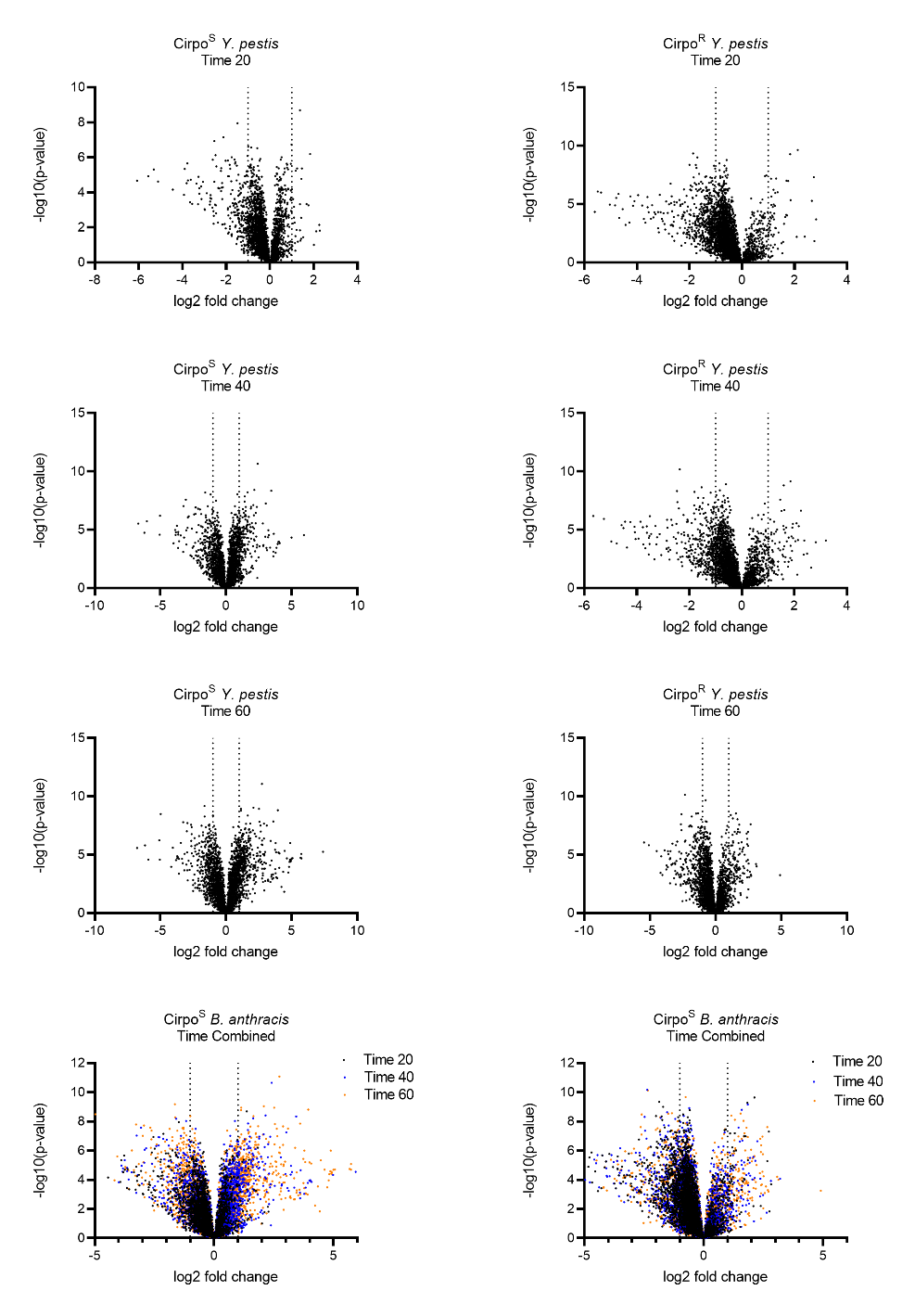


**Supplementary Figure S2: Transcriptomic analysis of Cipro^S^ and Cipro^R^ strains of *Y. pestis* in response to susceptible breakpoint concentrations of ciprofloxacin.** Volcano plots of RNA sequencing data showing log_2_ fold changes vs -log10(p-value) in Cipro^R^ and Cipro^S^ strains of *Y. pestis* across multiple timepoints after exposure to 0.25 µg/mL ciprofloxacin. Data is representative of RNA sequencing data from three biological replicates treated at each time point compared to time 0 untreated controls.


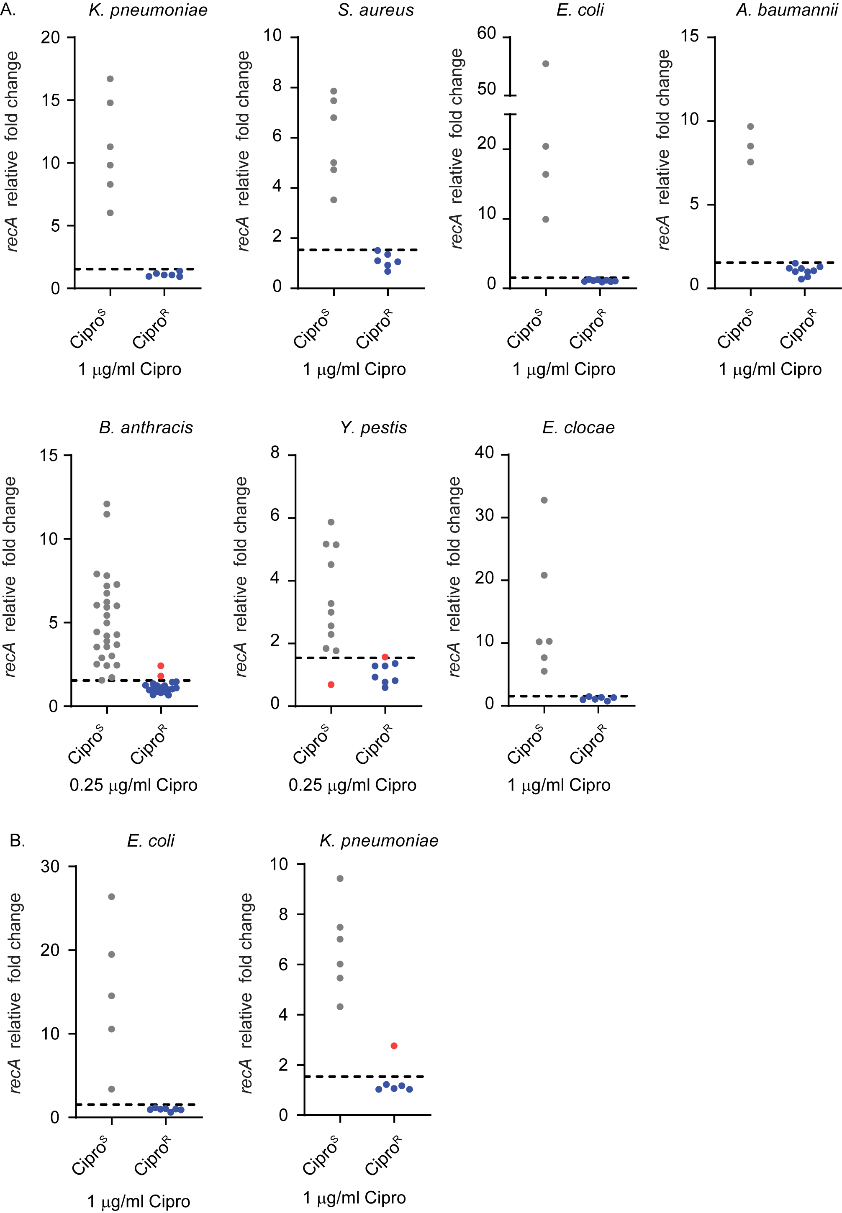


**Supplementary Figure S3: Assay performance utilizing *recA* fold changes to determine strain susceptibility.** Sensitive and resistant ESKAPE and biothreat pathogens grown overnight in A) culture media or B) urine were treated with indicated susceptible breakpoint concentrations of ciprofloxacin as determined by MIC interpretive criteria for each organism. Relative fold changes of recA were calculated for each strain using the ∆∆Cq method. Red circles represent strains which were called a major or very major error and subsequently repeated for confirmation. The average plus three standard deviations for each resistant strain within a species was calculated from Figure 3 and the greatest value, 1.54 was used as a cutoff to determine susceptibility.

**Supplementary Figure S4: Correlation of relative *recA* fold amongst strains and MICs.** A) Twelve strains of *K. pneumoniae* and *E. coli* were challenged with 1ug/mL ciprofloxacin after growth in broth or urine culture. Relative *recA* fold changes were measured using duplex RT-qPCR assays and plotted on X and Y axis for respective experimental conditions. B) XY plots correlating MICs to relative *recA* fold changes for Cipro^S^ and Cipro^R^ strains in response to breakpoint concentrations of ciprofloxacin.


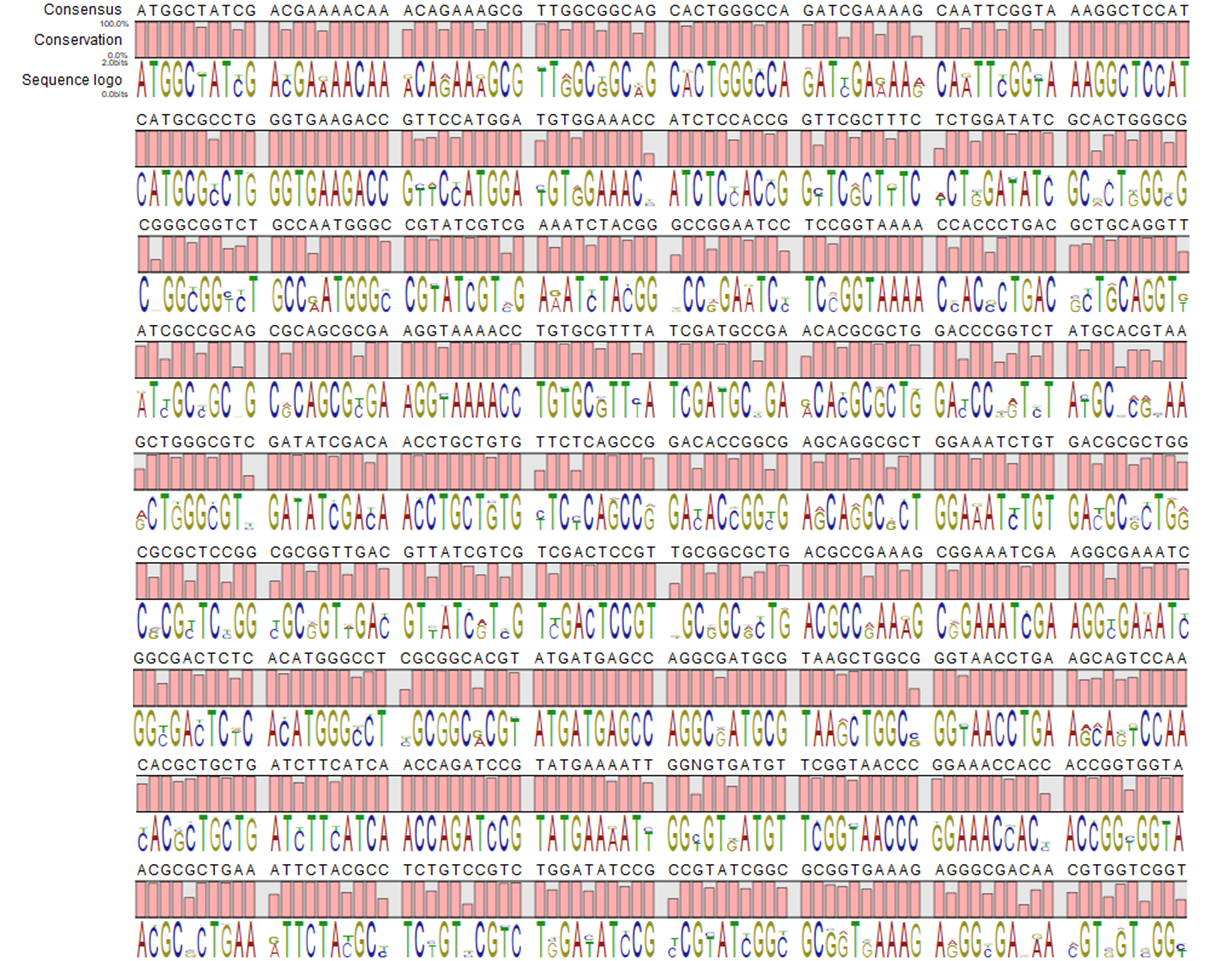


**Supplementary Figure S5: Alignment of recA gene for several members of the Enterobacteriaceae family.** *recA* genes from Enterobacteriaceae family members including *Citrobacter*, *Klebsiella*, *Morganella*, *Shigella,* *Escherichia*, *Enterobacter*, *Serratia*, and *Salmonella* were aligned using CLC genomics workbench. Consensus alignment of 86 total *recA* genes are shown for the first 720 bp.
